# TagSNP transferability and relative loss of variability prediction from HapMap to an admixed population

**DOI:** 10.1186/1423-0127-16-73

**Published:** 2009-08-14

**Authors:** Tulio C Lins, Breno S Abreu, Rinaldo W Pereira

**Affiliations:** 1 Programa de Pós-Graduação em Ciências Genômicas e Biotecnologia, Universidade Católica de Brasília, Brasília, DF, Brazil

## Abstract

**Background:**

The application of a subset of single nucleotide polymorphisms, the tagSNPs, can be useful in capturing untyped SNPs information in a genomic region. TagSNP transferability from the HapMap dataset to admixed populations is of uncertain value due population structure, admixture, drift and recombination effects. In this work an empirical dataset from a Brazilian admixed sample was evaluated against the HapMap population to measure tagSNP transferability and the relative loss of variability prediction.

**Methods:**

The transferability study was carried out using SNPs dispersed over four genomic regions: the PTPN22, HMGCR, VDR and CETP genes. Variability coverage and the prediction accuracy for tagSNPs in the selected genomic regions of HapMap phase II were computed using a prediction accuracy algorithm. Transferability of tagSNPs and relative loss of prediction were evaluated according to the difference between the Brazilian sample and the pooled and single HapMap population estimates.

**Results:**

Each population presented different levels of prediction per gene. On average, the Brazilian (BRA) sample displayed a lower power of prediction when compared to HapMap and the pooled sample. There was a relative loss of prediction for BRA when using single HapMap populations, but a pooled HapMap dataset generated minor loss of variability prediction and lower standard deviations, except at the VDR locus at which loss was minor using CEU tagSNPs.

**Conclusion:**

Studies that involve tagSNP selection for an admixed population should not be generally correlated with any specific HapMap population and can be better represented with a pooled dataset in most cases.

## Background

Since association studies were first introduced as a tool in understanding the genetic basis of complex phenotypes [1] an enormous methodological and analytical framework has been developed with regard to regions of high linkage disequilibrium (LD) and common haplotypes for genome-wide LD mapping [2,3]. The extension and localization of those regions are the mainstream in developing a set of SNPs capable of statistically representing untyped markers - the tagSNPs - reducing the costs of medium and high throughput genotyping in association studies [2-6]. The application of public genome data brought about great advances in the understanding of genetic variability and helped design association studies for complex phenotypes among several human populations of different ethnic backgrounds [7,8]. The three continental population samples in the HapMap project - Utah residents with northern and western European ancestry (CEU), East-Asians (Japanese from Tokyo and Han Chinese from Beijing) (CHB+JPT) and African Yoruba from Ibadan, Nigeria (YRI) - are used in experimental design as a reference for association studies in worldwide populations [6-9].

The challenge in establishing the HapMap as a standard for research is highlighted by the observation that the distribution of the haplotype blocks differs between population groups due to genetic and demographic effects [10]. However, tagSNP sharing from the HapMap dataset is commonly described as appropriately applied in European and East Asian populations [11-17], but less effective in other structured or multi-ethnic populations [9,10,18,19]. Such differences increase proportionally with the geographical distance between the HapMap data collection points and the actual sample collection [6,9,15,17]. Although the project never stated that these samples were representative of global variation, the fact that the HapMap study was carried out using only these ethno-geographic samples has been cited against the use of such data in populations that have a history of recent admixture [20-22].

Admixed populations can be useful in detecting genetic contributors to complex traits that differ in frequency between distinct populations. The admixture mapping approach has been proposed as an effective method for the identification of disease-susceptibility alleles with higher probability due to admixture-generated linkage disequilibrium extension [23]. Considering that the Latin-American people are one of the most heterogeneous around the world [24-26] as a result of mating primarily amongst three ethnic groups - Europeans, Native (South) Americans and Africans - the admixture mapping should be used as an alternative approach for the identification of disease-susceptibility loci [21,27].

Therefore, unintended use of tagging SNP data in admixed populations could lead to spurious results since there is evidence that admixture impacts the linkage disequilibrium structure, affecting the association of SNPs with etiological factors [28,29]. Such issues could render HapMap-based tagSNP selection approaches for admixed populations inaccurate or even useless. Moreover, knowledge of the degree of portability of HapMap data to admixed populations is also needed in order to comprehend whether there is loss or gain of variability when using tagSNPs selected from the consortium populations. Thus, the aim of this work was to develop a first approach to evaluate the tagSNP transferability from HapMap to the Brazilian admixed population, using 37 SNPs distributed between four loci: VDR, PTPN22, HMGCR and CETP.

## Methods

### Population sample

The sample of Brazilian subjects (BRA) consisted of 200 unrelated parents randomly selected from paternity test trios. A stratified sampling approach was adopted to represent the five Brazilian geopolitical regions according to each individual's place of birth. Genetic ancestry coefficients were estimated [30,31] so as to validate the admixture source of the population. All sampled individuals signed an informed consent allowing the use of their DNA for paternity testing and further anonymous population genetics research.

The genotypes of the HapMap population samples were retrieved from the database (Data Rel 21a/phaseII Jan07, on NCBI B35 assembly, dbSNP b125) consisting of 89 unrelated East Asian individuals (CHB+JPT) comprising 45 Han Chinese from Beijing and 44 Japanese from Tokyo; 90 individuals of northern and western European origin (CEU); and 90 Yoruba individuals (YRI) from Ibadan, Nigeria. All HapMap population genotypes for each gene were combined into a pooled sample (POOL; *n *= 269) in order to test a representative multi-ethnic population thereby resulting in a final set of five population samples: CHB+JPT, CEU, YRI, POOL and BRA. The research project was approved by the Universidade Católica de Brasília Ethics Review Board.

### SNP selection and genotyping

The SNP selection approach accounted for the markers that were polymorphic in at least one HapMap population and dispersed with average intervening distances of 5 kb [13,32]. Data for the HapMap analyses were dumped directly from the website (Table 1). Genotyping in the Brazilian sample was performed using an optimized PCR reaction to co-amplify the fragments in distinct multiplex panels for each gene marker. Afterwards, the PCR-amplified products were purified by enzymatic treatment with exonuclease I (ExoI) and shrimp alkaline phosphatase (SAP) enzymes in order to eliminate non-incorporated dNTPs and primers. Finally, the minisequencing reaction was performed using the SNaPshot^® ^Multiplex minisequencing kit reaction mix (Applied Biosystems) and the products of the SNaPshot^® ^reaction were analyzed on the ABI 3100 Genetic Analyser (Applied Biosystems) using an ABI 3700 POP-6^© ^polymer. Genotypes were called using GeneScan Analysis Software, version 3.7 (Applied Biosystems) and Genotyper version 3.7 (Applied Biosystems). An optimized multiplex single-base extension PCR was implemented according to a protocol described elsewhere [33].

**Table 1:** Characteristics of genomic regions genotyped in this study

SNP rs	Gene	Allele	Chr	Position	Average Distance (Kb)	Gene Extension (Kb)
rs3789607	PTPN22	C/T	1	114078476	5.80	34.80
rs2476600	PTPN22	A/G	1	114081776		
rs1217395	PTPN22	A/G	1	114086477		
rs2476601	PTPN22	A/G	1	114089610		
rs2476602	PTPN22	A/G	1	114108997		
rs1217418	PTPN22	A/G	1	114113273		
rs3931914	HMGCR	C/G	5	74663770	4.08	28.52
rs3761740	HMGCR	A/C	5	74667889		
rs10515198	HMGCR	C/T	5	74677316		
rs2241402	HMGCR	A/T	5	74682011		
rs12654264	HMGCR	A/T	5	74684359		
rs2303151	HMGCR	A/G	5	74691207		
rs12916	HMGCR	C/T	5	74692295		
rs2544040	VDR	A/G	12	46509213	4.42	79.60
rs11608702	VDR	A/T	12	46515035		
rs7968585	VDR	C/T	12	46518360		
rs9729	VDR	A/C	12	46522890		
rs731236 (TaqI)	VDR	C/T	12	46525024		
rs7975232 (ApaI)	VDR	A/C	12	46525104		
rs1544410 (BsmI)	VDR	A/G	12	46526102		
rs2248098	VDR	C/T	12	46539623		
rs2239179	VDR	A/G	12	46544033		
rs886441	VDR	C/T	12	46549231		
rs10735810 (FokI)	VDR	A/G	12	46559162		
rs2254210	VDR	A/G	12	46559981		
rs2853564	VDR	C/T	12	46564754		
rs2853559	VDR	C/T	12	46569072		
rs3890734	VDR	A/G	12	46575622		
rs10783219	VDR	A/T	12	46581755		
rs4516035	VDR	C/T	12	46586093		
rs11568820 (CDX-2)	VDR	A/G	12	46588812		
rs3764261	CETP	G/T	16	55550825	5.10	30.61
rs711752	CETP	A/G	16	55553712		
rs1532624	CETP	G/T	16	55562980		
rs5882	CETP	A/G	16	55573593		
rs2303790	CETP	A/G	16	55574793		
rs289747	CETP	A/G	16	55581439		

SNPs are identified by their rs number, gene, alleles, chromosome and position according to HapMap release #23a (NCBI build 36, dbSNP b126), average distance between markers and gene extension in Kb.

### TagSNP transferability and LD analysis

The tagSNP transferability study was conducted using the Stampa algorithm [34] implemented on the Gevalt package [35]. This algorithm aims to maximize the expected accuracy of predicting untyped SNPs based on genotype data of the tagSNPs [34]. To conduct this study, first the variability prediction accuracy for each gene was assessed to calculate the coverage of the HapMap phase II data in relation to the total number of available SNPs in each region: number of common SNPs - with minor allele frequency (MAF) > 0.05; number of SNPs required to capture 100% of SNP prediction; maximum prediction using the same number of SNPs as in the study; and the prediction for the selected set of SNPs. Then, the set of SNPs selected with average distances of 5 Kb had their variability prediction calculated based on two until the maximum number of tagSNPs for all five samples. Finally, the relative loss of variability prediction (in percentage points; pp) was calculated by subtracting the variability prediction of tagSNPs selected for BRA from the relative prediction obtained when using the tagSNPs selected for each of the HapMap populations and the pooled sample in the Brazilian group.

Measures of linkage disequilibrium (LD) between pairs of SNP loci (*D' *and *r*^2^) were calculated by the Gerbil algorithm [36], implemented in Gevalt, using the standard maximum-likelihood and expectation-maximization algorithm methods. Only the SNPs accounted for in all five populations were evaluated. A pairwise population LD analysis was carried out using a Spearman's correlation coefficient.

## Results

### Variability coverage of HapMap

The characteristics of each gene region varied according to the number of SNPs available in phase II of HapMap (Table 2). The most critical difference was the SNP density at each region, which varied from approximately 0.80 to 3.30 SNPs per Kb, though it was conserved among populations (Table 2). The overall average variability of the selected SNPs was 89.55% representing 6.7 percentage points (pp) of loss from the maximum of variability using the same number of tagSNPs selected by the algorithm. Each population presented a different loss of prediction per gene. The population average that presented the highest loss of prediction was CHB+JPT with 8.11 pp, followed by CEU (7.33 pp) and YRI (4.68 pp). The gene that had the highest loss of prediction on average was the PTPN22 (9.40 pp), followed by CETP (7.33 pp), HMGCR (5.21 pp) and VDR (4.87 pp).

**Table 2:** SNP prediction Coverage for HapMap population samples

Population	Gene	Total SNPs	Density (SNP/Kb)	Common SNPs	n to 100% prediction	Prediction of selected SNPs (%)	Max. Prediction (%)
CHB+JPT	PTPN22	30	0.86	21 (70%)	14	87.15	99.59
CEU		28	0.80	21 (75%)	17	88.00	98.50
YRI		29	0.83	17 (59%)	12	93.91	99.17
							
CHB+JPT	HMGCR	23	0.81	20 (87%)	19	90.17	98.12
CEU		24	0.84	22 (92%)	21	93.56	97.50
YRI		23	0.81	17 (74%)	16	94.33	98.08
							
CHB+JPT	VDR	162	2.04	84 (52%)	73	92.91	98.00
CEU		161	2.02	94 (58%)	79	92.12	96.67
YRI		159	2.00	107 (67%)	101	88.20	93.17
							
CHB+JPT	CETP	102	3.33	55 (54%)	51	86.18	93.13
CEU		101	3.30	48 (48%)	40	82.74	93.07
YRI		102	3.33	69 (68%)	61	85.33	90.05

Coverage is based on a total number of SNPs, region density measured by SNP/Kb, number of common SNPs (MAF <0.05), number of common SNPs to reach 100% of prediction, prediction of the selected SNPs and the maximum prediction using the same number of SNPs.

**Figure 1:**
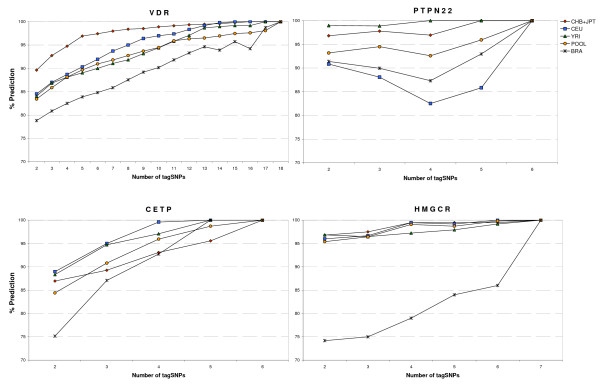
**Variability prediction in each gene**. Percentage of prediction is described in each population sample from the minimum of two to the maximum number of SNPs studied in each loci (VDR, PTPN22, CETP and HMGCR).

The prediction power of the evaluated SNPs differed among the genes. Overall, the Brazilian sample displayed a lower power of prediction when compared to HapMap and the pooled sample. The only exception occurred in the PTPN22 gene where CEU predictions were always lower than those for BRA (Figure 1). At the HMGCR gene, the prediction was, on average, 15.34 pp lower for BRA than the average for the other HapMap populations (Figure 1), while in other genes this difference was smaller (VDR 5.36 pp, PTPN22 3.32 pp and CETP 3.92 pp).

### TagSNP transferability analysis

To evaluate the transferability of tagSNPs, the prediction of variability coverage in the BRA sample was calculated for the set of SNPs in each of the HapMap populations and the POOL sample. The relative loss was calculated by subtracting the prediction coverage using the HapMap tagSNPs from the prediction coverage of those tagSNPs in BRA. This simple calculation gives an idea of the prediction loss as opposed to a true prediction in an admixed sample, since the SNPs evaluated are presented for all population data. The average prediction loss varied among genes and among populations (Table 3). Considering only the HapMap samples, CHB+JPT had the lowest prediction loss on average, followed by CEU and YRI, but in general, the pooled HapMap sample resulted in the lowest relative prediction losses (Table 3). When using only one population tagSNP as reference there can be substantial losses in some regions, for instance the VDR and PTPN22 genes when using YRI tagSNP, while in other cases there can be minor loss, as observed in the HMGCR gene when using YRI tagSNPs. It was observed that the loss of prediction tends to increase as the number of tagSNP increases, but decreases or becomes stable with the last groups of tags (data not shown).

**Table 3:** Loss of SNP prediction coverage in BRA using HapMap tagSNPs

TagSNP set	PTPN22	HMGCR	VDR	CETP	A.V. ± S.D.
CHB+JPT	3.035	4.936	6.448	0.253	3.668 ± 2.671
CEU	7.995	3.600	4.211	2.120	4.481 ± 2.501
YRI	7.608	2.222	12.078	3.580	6.372 ± 4.438
POOL	2.940	4.390	4.221	2.198	3.437 ± 1.050

Loss of SNP prediction coverage is given by percentage point difference between the SNP prediction defined in BRA and the prediction in BRA using the set of tagSNPs selected for the HapMap populations. Last column displays average values ± standard deviation.

### Pairwise LD analysis

A comparison of pairwise LD correlation analysis was assessed between the Brazilian sample, the HapMap and the pooled data. When each region was examined individually, LD analysis between BRA and the other samples did not find significant values for *D' *measurements (data not shown), except for at the VDR locus, for which Spearman's correlation coefficients (rho) were 0.067 for YRI, 0.401 for CHB+JPT, 0.737 for CEU and 0.632 for POOL, whereas for LD *r*^2 ^a higher correlation was found for the POOL data, except for at the VDR locus (Table 4). When all pairs of SNPs were compared between BRA and the other populations the correlation coefficients followed the same order using either *D' *or *r*^2 ^(CEU, POOL, CHB+JPT), and LD *r*^2 ^correlation coefficients (rho) were slightly higher when compared to *D' *measurements.

**Table 4:** Spearman's correlation coefficient (rho)

*r*^2^	CHB+JPT	CEU	YRI	POOL
PTPN22	0.697	0.813	0.543	0.949
HMGCR	0.853	0.816	0.862	0.902
VDR	0.312	0.742	0.321	0.639
CETP	0.821	0.785	-0.018*	0.912
overall	0.491	0.782	0.431	0.719

Correlation of LD measures (*r*^2^) for all SNP pairs between BRA and HapMap populations. * p-value not significant, all others were statistically significant (*p *< 0.05).

## Discussion

The success of a genetic association study is strongly affected by marker selection for a specific population. With regard to admixed populations this criterion is of fundamental concern due to the risk of spurious associations in the case of inefficient choice. The HapMap Consortium provided solutions for most cases by making available millions of markers genome-wide that were genotyped in each of the continental populations, although it did not address how markers selected in one or more HapMap samples will perform in studies with other populations [8]. To date, several studies have evaluated tagSNPs portability in a range of worldwide populations, but none has assessed a heterogeneous admixed population. The present study indicates that tagSNP sets from HapMap population can be portable to admixed populations to a reasonable degree, however the results can also be uncertain and inaccurate if applied improperly. It also demonstrates the necessity for understanding the patterns of physical (gene extension and SNP density) and genetic (LD patterns) differences in every genomic region prior to determining the tagSNPs to be used, in order to make a reasonable prediction for untyped markers.

Measures of LD and SNP density vary across the genome and can be critical points when selecting a set of tagSNPs. A study by Tantoso and colleagues [37] showed that SNPs can be transferred from HapMap to other populations of the same ethnic and continental origin. Even so, tagSNP coverage increases along with the SNP density due to the high LD in European and the Asian populations. Hence, coverage of many untyped variants, especially the rare ones (MAF <0.05), drops from 50% to 30% depending on the population used [37]. Another study [15] showed that the SNP density has a major effect on tag selection, proposing denser sets (i.e., one SNP every 1.3 kb) to improve the tagSNP performance. In the present study the SNPs were selected with SNP density that was approximately equal in the four regions studied (one SNP every 5 kb), to reduce or eliminate such an effect. Using the same genotyped SNP density at two regions with physically different densities - CETP (30 kb and 3.3 SNP/kb) and HMGCR (28 kb and 0.8 SNP/kb) - demonstrates that either maximum or minimum prediction among regions and within the population provided no more than 10 percentage points of loss in prediction (Table 2). Though, the fact that the prediction becomes stable or decreases as the number of tagSNPs increases is evidence that SNP density can be a critical point in tagSNP selection in larger genome-wide sets [15,37], as well as in low-throughput region analysis, emphasizing that for an admixed population it is necessary to use, in a reduced panel, as many SNPs as possible.

The SNP prediction and tagSNP transferability are also dependent on the linkage disequilibrium patterns and hence in admixed populations they can be influenced both by the demographic events and by genetic factors. Generally, tagSNP sets selected for similar populations with similar haplotype block structures have better performance but differ if the block structures and boundaries also differ [6,9-12,38]. For instance, CEU tags are useful for populations with European ancestry and tagSNPs selected for YRI perform well in Sub-Saharan Africans, but require larger genotype densities due to lower LD among markers [11,12,37].

The linkage disequilibrium measures could be evidence leading to the belief that one could use tagSNPs directly transferred from CEU to BRA without great loss of variability, since the greatest ancestral contribution in the Brazilian sample is European [24,25,30,31]. Considering all SNP pairs in the current dataset the pairwise LD had the highest correlation between BRA and CEU, followed by POOL, CHB+JPT and YRI, which had the lowest average LD and was less correlated. However when genes were analyzed individually, except for the VDR gene, the POOL data had the highest correlation compared to the other populations.

Although using tagSNPs directly form CEU worked with great efficiency in some cases, as in the case of VDR gene, in others this type of selection provided greater loss of variability, as in the specific case of the PTPN22 gene, reinforcing the idea that each genomic region will perform according to gene and population structure [6]. Linkage disequilibrium arising from the recent admixture of genetically distinct populations can be categorized as a genome-wide effect and thus selecting markers from representative parental populations offers analytical risks due to the fact that in some genomic regions, particularly those with high LD, ancestral haplotype-block structures at the individual level are not always eliminated by recent admixture.

Population stratification along the Latin American populations varies extensively as consequence of their history of immigration and colonization over the last five centuries. In Brazil there is a major contribution from the European ancestry followed by African and Amerindian [24,25,30,31]. In the present data the pooled sample tagSNP performance had a relative loss of prediction smaller than any other population sample. Although the relative loss of prediction among CHB+JPT and POOL were very close, the fact that standard deviation in the pooled sample was lower demonstrated that, in a study with multiple gene analysis, it can be a safe alternative to choose tagSNPs from the pooled samples, because different LD patterns at different genes can have different SNP coverage depending on each of the HapMap populations [6].

In other Latin-American populations, such as those from Mexico or Argentina, the contributions of the Amerindian proportion at population level are usually higher than in Brazil, and African ancestry is higher in Caribbean populations than in any other [39-41]. Such population structure difference should be considered when applying a tagSNP selection method depending on each specific case of admixture. It is possible that for Mexicans or Argentineans a combination of the CEU, CHB and JPT HapMap samples would perform better than the whole HapMap pool, as was the case for South Asian populations such as the Indian population [6] and Hazara, Kalash and Uygur populations [11]. The combinations of HapMap panels were also effective at representing other populations, such as the Philippines [42], for which CHB samples and the combined CHB+JPT samples were most transferable to Cebu Filipino samples, indicating that different pools of HapMap panels should be tested and used as an alternative in many situations.

However, it is noteworthy that the SNP coverage in HapMap is not complete and tagging strategies critically depend on the investigation of other population polymorphisms [18]. The project is now overcoming the representative world-wide population issue with the Phase III release, which includes Amerindian and Mexican ancestral populations among others. This will certainly improve the methods of tagSNP selection for admixed populations but a comprehensive study using high-throughput genome-wide SNPs in assorted admixed populations will be required to reduce confounding effects caused by population stratification and to enhance the tagSNP performance. Identification, re-sequencing, and genotyping of large-scale and high-throughput SNP data were beyond the scope of this study. Further analysis will be necessary to assess if such techniques will attain the same level of efficiency in other admixed populations in which a history of admixture processes differs from the Brazilian sample, known for being recent and continuous, as opposed to populations which have undergone well defined time limited admixture processes in the past.

## Conclusion

The pooled HapMap sample provided the minimum loss of prediction in admixed population and therefore, combined with the SNP selection spaced at most every 5.0 kb may represent an efficient alternative. The present findings will be useful for the future design and analysis of genetic studies using other admixed populations, suggesting that on such occasions the selection of markers should not be generalized according to the tagSNPs of one or other current HapMap populations due to genetic and demographic effects.

## Competing interests

The authors declare that they have no competing interests.

## Authors' contributions

TCL performed molecular analysis of PTPN22 and VDR genes, interpreted the data and drafted the manuscript. BSA designed the study, performed molecular analysis of CETP and HMGCR genes, interpreted the data and participated in manuscript drafting. RWP conceived, coordinated and designed the study. The authors read and approved the final manuscript.
